# The Era of Radiogenomics in Precision Medicine: An Emerging Approach to Support Diagnosis, Treatment Decisions, and Prognostication in Oncology

**DOI:** 10.3389/fonc.2020.570465

**Published:** 2021-01-26

**Authors:** Lin Shui, Haoyu Ren, Xi Yang, Jian Li, Ziwei Chen, Cheng Yi, Hong Zhu, Pixian Shui

**Affiliations:** ^1^ Department of Medical Oncology, Cancer Center, West China Hospital, Sichuan University, Chengdu, China; ^2^ Department of General, Visceral and Transplantation Surgery, University Hospital, LMU Munich, Munich, Germany; ^3^ Department of Pharmacy, The Affiliated Traditional Chinese Medicine Hospital of Southwest Medical University, Luzhou, China; ^4^ Department of Nephrology, Chengdu Integrated TCM and Western Medicine Hospital, Chengdu, China; ^5^ School of Pharmacy, Southwest Medical University, Luzhou, China

**Keywords:** precision medicine, deep learning, artificial intelligence, radiogenomics, radiological imaging

## Abstract

With the rapid development of new technologies, including artificial intelligence and genome sequencing, radiogenomics has emerged as a state-of-the-art science in the field of individualized medicine. Radiogenomics combines a large volume of quantitative data extracted from medical images with individual genomic phenotypes and constructs a prediction model through deep learning to stratify patients, guide therapeutic strategies, and evaluate clinical outcomes. Recent studies of various types of tumors demonstrate the predictive value of radiogenomics. And some of the issues in the radiogenomic analysis and the solutions from prior works are presented. Although the workflow criteria and international agreed guidelines for statistical methods need to be confirmed, radiogenomics represents a repeatable and cost-effective approach for the detection of continuous changes and is a promising surrogate for invasive interventions. Therefore, radiogenomics could facilitate computer-aided diagnosis, treatment, and prediction of the prognosis in patients with tumors in the routine clinical setting. Here, we summarize the integrated process of radiogenomics and introduce the crucial strategies and statistical algorithms involved in current studies.

## Background

Advances in genomics and the far-reaching effects of precision medicine have synergistically accelerated research by integrating the individual characteristics of patients (1). Compared with conventional medical treatment, the concept of precision medicine follows a “one-size-fits-one” philosophy and sets out a tailored therapeutic plan according to the genotypic and phenotypic data of individual patients (2).

Cancer is a disease that involves genetic abnormalities caused by hereditary or environmental factors. When genes undergo the error-prone process of replication and alterations, such as nucleotide substitution, insertions, deletions, and chromosomal rearrangements, the activation of oncogenes and loss of tumor suppressor genes may induce oncogenesis (3). Moreover, epigenetic alterations, including histone modification, DNA methylation, and altered expression levels of non-coding RNAs, have also been confirmed to be important contributors to the development of cancer (4). Over recent decades, there have been major advances in our understanding of the genetic alterations involved in oncogenesis. For example, mutations of the Kirsten rat sarcoma viral oncogene (*KRAS)*, epidermal growth factor receptor (*EGFR*), and anaplastic lymphoma kinase (*ALK*) genes have been identified to be common oncogenic drivers (5). These abnormalities of specific molecular and signaling pathways can be used as genomic biomarkers that provide personalized information about diagnosis, treatment, and prognosis, and contribute to selection of the optimal therapeutic strategy.

Access to genomic information in conventional clinical procedures is based mainly on biopsy of focal tissue samples and microarray genetic analysis. Histopathological examination is feasible to decipher mutational signatures and genomic information, but these data can only reflect the status of a tumor at the time of biopsy or resection. Moreover, gene expression profiling of only a fraction of the tumor tissue cannot reflect the heterogeneity of the entire tumor. The spatial and temporal variables of gene expression may cause changes in various biological processes in the tumor, including apoptosis, cellular proliferation, growth patterns, and angiogenesis. These alterations occur at the molecular and cellular levels and, to a large extent, are shown as heterogeneous imaging features, which can be transformed into varying degrees of signals in different imaging platforms using radiological technology (6).

Technological progress in microarrays, automated DNA and RNA sequencing, mass spectrometry, and comparative genomic hybridization are essential for exploration of tumor biomarkers and more accurate assessment of disease status in patients, as shown in pancreatic cancer (7). Nowadays, large databases that are suitable for elucidating the relationship between gene expression and clinical features exist. When combined with artificial intelligence, treatment options and survival could be predicted by the performance of individuals in models based on big data (8). Currently, non-invasive detection and monitoring of diseases can be performed repeatedly without causing harm and has become a hotspot in cancer research. The huge diversity of phenotypes can be demonstrated by non-invasive radiological imaging (9), which reveals many characteristics of tumors in both subjective and qualitative ways (10). Recent advances in image acquisition, standardization, and image analysis have allowed identification of objective and precise imaging features, including prognostic and predictive biomarkers (11). Although imaging examinations are often performed repeatedly during treatment, it is still impractical to obtain dynamic genomic or proteomic data. Fortunately, this problem can be solved by analyzing computer-processed images to find underlying predictive and prognostic information (12). Radiomics refers to the qualitative and quantitative extraction of data from clinical images and clinical information as well as the methodology used to convert these features in a way that supports decision-making. Radiogenomics, a new computational discipline, is an emerging area within radiomics and is a combination of the words “radiology” and “genomics” (13). The advent of radiogenomics reflects a shift in the focus of radiology-pathology research from the gross anatomical level to the genetic level. Moreover, radiogenomics aims to investigate the correlation between the integrated hierarchical analysis of a massive number of imaging characteristics and corresponding gene expression profiles and to identify optimal radiomic biomarkers, so as to allow more reliable prediction of prognosis and response to treatment.

## Advances in Methodology, Technology, and Workflow in Radiogenomics

The enormous amount of imaging data collected has resulted in a large-scale database that is both diverse and complex. Therefore, advanced frameworks, techniques, algorithms, and analytics are needed to mine significant and valuable radiomic information from the imaging database (8).

The birth and development of radiogenomics relies on high-throughput computing and machine learning, both of which are good methods for managing and analyzing a very large number of variables for different samples and procedures. For example, although the image of a tumor region is typically assessed by a radiologist using functional or morphological features, the imaging actually contains more complicated information that can be extracted and processed effectively using radiogenomic approaches.

**Figure 1:**
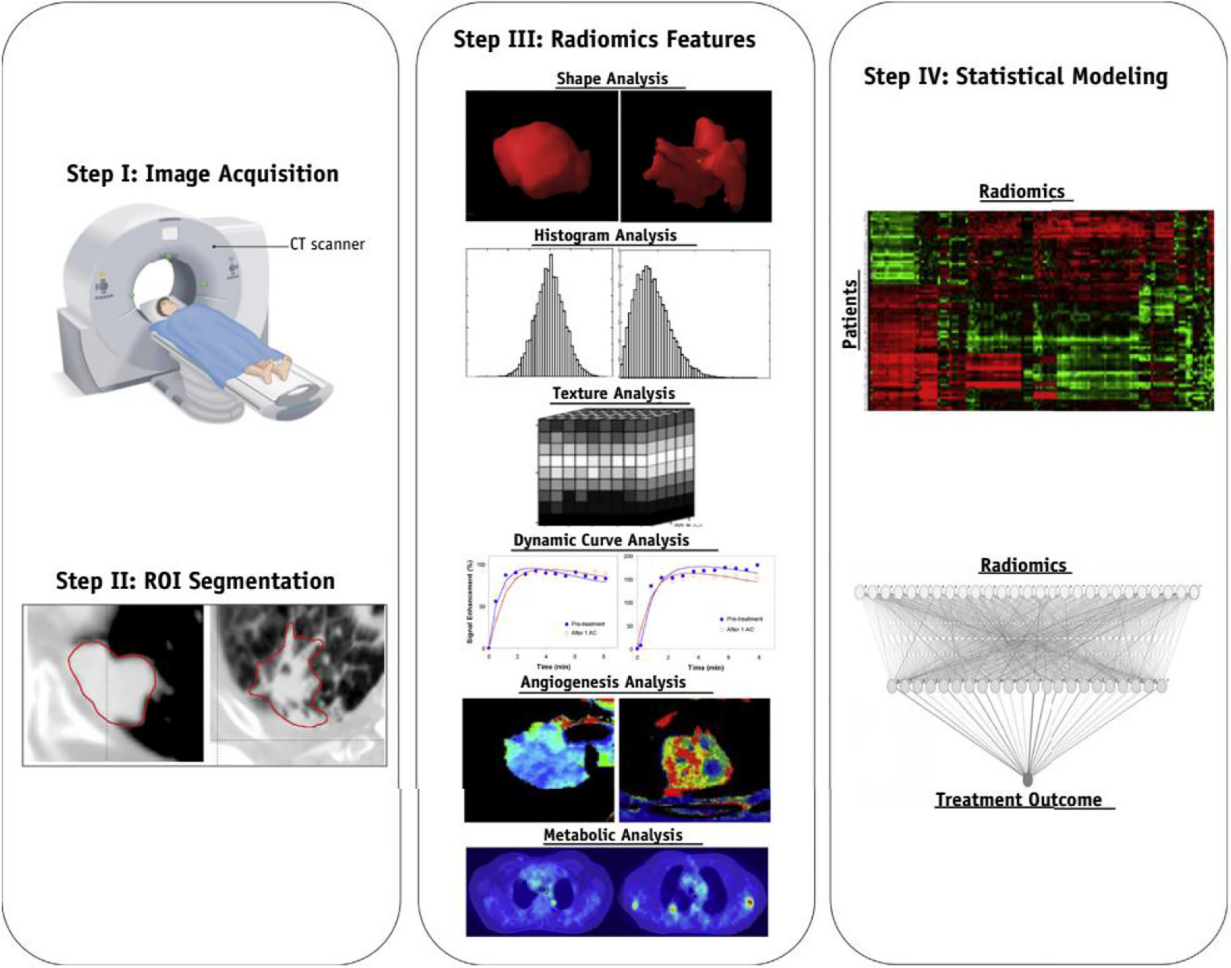
The general radiomics study workflow. Step 1: image acquisition. Step 2: region of interest identification and segmentation. Step 3: quantitative image feature extraction. Step 4: data mining and informatics analysis. The figure was reprinted by ref (14) with permission from the publisher.

The workflow of radiogenomics mainly includes data acquisition and pre-processing, tumor segmentation, feature extraction, analysis, and modeling (**Figure 1**). The standardized operating procedure of radiogenomics is a basic assurance of the quality of studies to reduce avoidable errors, particularly in multicenter studies. Along these lines, the Image Biomarker Standardization Initiative consortium, standardized 169 radiomics features in two phases, most of which showed high reproducibility in the third phase and can be applied in different radiomics software (15). Below we provide a brief overview of a feasible imaging protocol for radiogenomics.

## Development of Radiomics Prediction Models

### Acquisition of Raw Images

In oncology, multimodality imaging, such as positron emission tomography (PET)-computed tomography (CT) and single-photon emission CT, can describe both the anatomical and functional features of tumors in great detail. However, recent efforts have focused on a combination of quantitative functional assessments, such as multiple PET tracers, various magnetic resonance imaging (MRI) contrast mechanisms, and PET-MRI, thereby revealing multidimensional features of the tumor phenotype (16–18). For instance, diffusion-weighted MRI is capable of reflecting tumor density and cellularity, and can therefore be used to monitor the response to cytotoxic treatment (19). Furthermore, fluorodeoxyglucose (FDG)-PET is a molecular imaging tool that is frequently used to characterize changes in metabolic activity within a tumor. The rate of uptake, metabolism, and accumulation of FDG can be used to assess the therapeutic effects and disease progression (16, 20, 21). Different parameters can be acquired using different radiological imaging technologies. Therefore, selection of imaging equipment or technology is important for acquisition of desirable parameters.

### Pre-Processing of Information

Raw imaging data need to be pre-processed in order to maintain homogenous and reliable traits. One optional step is filtering the imaging signals within the region of interest (ROI). Manual segmentation is the most widely used method but requires clinicians to have sufficient experience to be able to delineate the optimal ROI. If the ROI is too small, it cannot provide sufficient information about voxels for analysis, and if it is too large, it may be easily biased by the heterogeneity of the tumor. However, full manual segmentation may have some limitations, being time-consuming and showing inter-observer variability (22, 23). Although automatic segmentation is superior to manual delineation in terms of precision and efficiency, its performance depends on the accuracy of the algorithm used and its ability to differentiate ROIs from surrounding tissues.

The critical issues of the robustness of quantitative features with respect to imaging variations and inter-institutional variability need to be investigated further. Currently, there are several advanced machines equipped with deep learning-based algorithms aimed at contour functions, including the 3DSlicer (24–26), DeepMind (Google) (27), and Project InnerEye (Microsoft) (https://www.microsoft.com/en-us/research/project/medical-image-analysis/).

An increasing number of studies have proven that the preferred mode for imaging pre-processing is semi-automatic segmentation, which makes use of both manual intervention and software automation (28). Tixier et al. (29) compared the robustness of 108 radiomic features from five categories using a semi-automatic and an interactive segmentation method by two raters. The results demonstrated that the interactive method produced more robust features than the semi-automatic method; however, the robustness of the radiomic features varied by categories. Um et al. (30) used five image pre-processing techniques: 8-bit global rescaling, 8-bit local rescaling, bias field correction, histogram standardization, and isotropic resampling to extract a total of 420 features from 161 cases. Of the examined techniques, histogram standardization was concluded to contribute the most in reducing radiomic feature variability, since it was shown to reduce the covariate shift for three feature categories and to be capable of discriminating patients into groups based on their survival risks. Veeraraghavan et al. (31) developed a novel semi-automatic approach that combines GrowCut (GC) with cancer-specific multiparametric Gaussian Mixture Model (GCGMM) to produce accurate and reproducible segmentations. Segmentation performance using manual and GCGMM segmentations was compared in a sample of 75 patients with invasive breast carcinoma. GCGMM’s segmentations and the texture features computed from those segmentations were shown to be more reproducible than manual delineations and other analyzed segmentation methods.

### Extraction of Features

**Figure 2:**
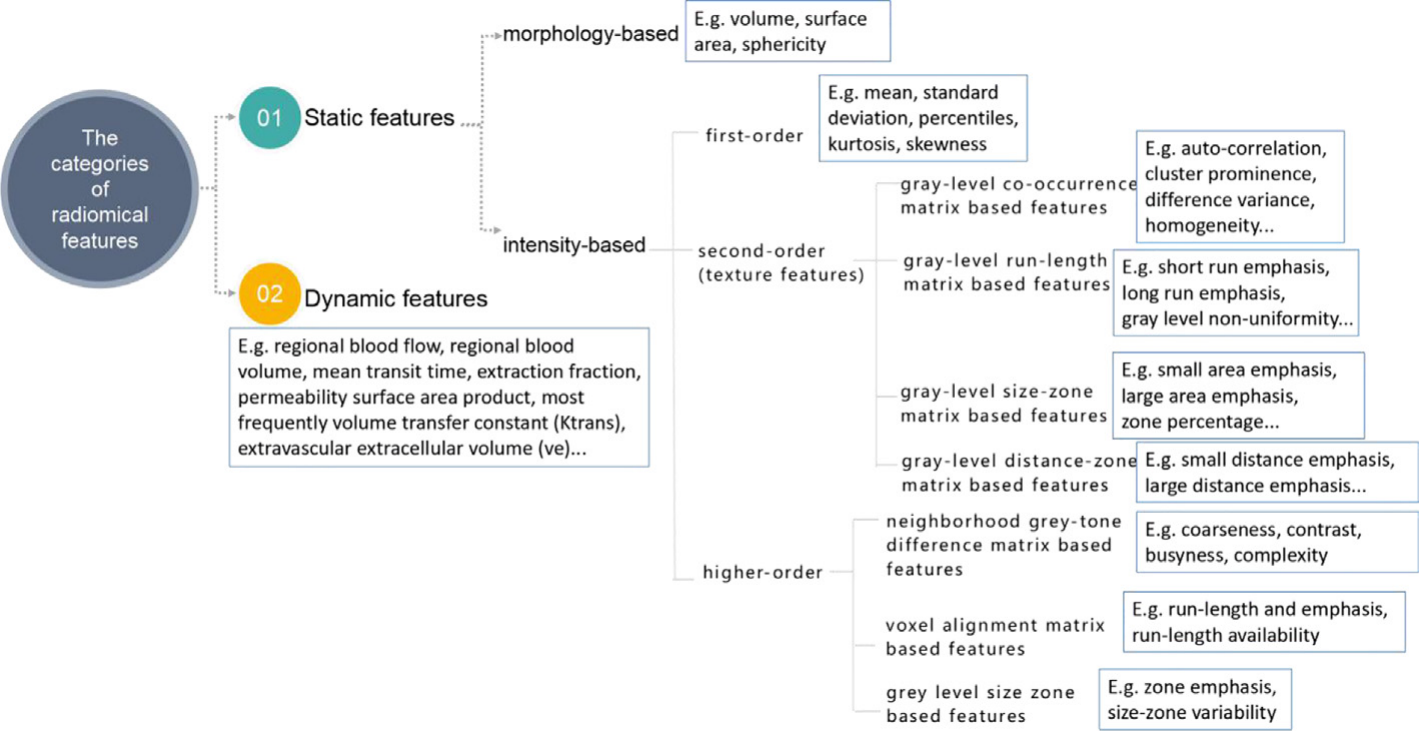
The classifications and corresponding examples of quantitative radiomics features. The figure was reproduced according to ref (14). with permission from the publisher.

The critical component of radiomics is the extraction of high-dimensional feature sets to quantitatively describe the attributes of oncological phenotypes. These extracted quantitative data reflect the crucial part of the establishment of radiomics prediction models. In practice, 50 to 5,000 radiomic features processed by specific software, including PyRadiomics (32, 33), CERR (34, 35) or IBEX (36, 37), are usually divided into morphological, intensity-based, and dynamic features (14) (**Figure 2**). Morphology-based features can collect three-dimensional (3D) shape characteristics, including volume, surface area, and sphericity. Intensity-based features can evaluate the gray-level distribution inside the ROI, which can characterize the overall variability in intensity (first-order) and the local distribution (second-order, also referred to as “texture features”). In terms of oncological pathology, both tumors and precancerous lesions have highly heterogeneous cell populations with normal stromal and inflammatory cells. Compared with conventional pathology, which only reveals underlying biological information in subregions, advanced texture analysis is emerging as a novel medical imaging tool for assessment of intratumoral heterogeneity. Texture analysis is used to describe the association between the gray-level intensity of pixels or voxels and their position within ROIs. Texture analysis usually consists of four steps: extraction, texture discrimination, texture classification, and shape reconstruction. Moreover, previous studies have demonstrated that non-uniform staining intensity within tumors may predict more aggressive behavior, poorer response to treatment, and worse prognosis (14, 38).

Furthermore, dynamic features derived from dynamic contrast-enhanced CT or MRI and metabolic PET (which can be one or more voxels within the ROI) are widely used to quantify enhancement of or uptake in tumors over time. Evaluating these extracted dynamic features can uncover relationships with molecular subclassifications of tumors and the prognosis (39).

An even more extensive range of features is expected. These radiomics features provide additional data associated with tumor pathophysiology that cannot be achieved by typical radiological interpretation. Therefore, creation of more easily interpretable models will be key to establishing a correlation between radiography and radiomics features in the future. Moreover, the stability and accuracy of features should be validated by test-retest datasets, and any that are volatile or unreliable should be excluded.

### Data Analysis

The variables and features collected during extraction are often redundant and may contain unnecessary information that leads to overfitting. Therefore, selection or dimensional limitation of the basic data is essential to maintain the selected significant imaging characteristics that show a strong correlation with clinical events.

The common selection methods can be classified into three categories: filter, wrapper, and embedded methods. Groups of highly correlated radiomics features can be identified *via* clustering. Filter methods evaluate features without involving the model in a univariate or multivariate way, which means the rank criterion depends only on the relevance of the feature or use of a weighted sum to maximize relevance and minimize redundancy. Features can then be generated and evaluated using the model with wrapper methods. Finally, a feature subset is proposed and evaluated during construction of the model with embedded methods.

#### Deep Learning and Convolutional Neural Networks

Deep learning is a machine learning algorithm that is characterized by utilization of neural networks with multiple layers (40). It is regarded as a semi-theoretical and semi-empirical modeling method that can be used to construct a holistic architecture on the basis of mathematical knowledge or computing algorithms, correlate training data to large-scale computing ability, adjust internal parameters, and consequently solve target problems. Convolutional neural networks (CNNs) are typically used in deep learning and combine imaging filters with artificial neural networks through a series of successive linear and nonlinear layers (41). CNNs use local connections and weights to analyze the input images, followed by pooling operations to obtain spatially invariant features (42). Furthermore, a fully connected network created at the end of the CNN could convert the final two-dimensional layers into a one-dimensional feature vector (43). After obtaining sufficient training data, deep learning algorithms can determine the optimal feature set and the relative importance of each feature. They can then classify images by utilizing combinations of features. Therefore, machine learning has become a fitting approach for selection and classification of features (44).

### Outcome Modeling Through Machine Learning

Once the feature set is obtained, a prediction model is needed to connect the features selected with the genetic information of the diseases in order to prospectively identify subgroups of patients who may benefit from specific treatment. However, without interpretability, these quantitative descriptors are inconvenient and difficult to apply when using radiogenomics in clinical practice. Therefore, interpretable models are required to establish correlations between quantitative formula-derived radiomics features and genetic subtypes. Representative classification methods include conventional logistic regression (45) and advanced machine learning techniques (46), such as decision trees and random forests, support vector machines, and deep neural networks (47), which are able to emulate human intelligence by acquiring knowledge of the surrounding environment from the input data and detect nonlinear complex patterns in the data.

Machine learning can build prediction models in several ways and includes unsupervised, supervised, and semi-supervised approaches. Unsupervised analysis divides the data into subgroups based on the similarity between samples. In the unsupervised model, a distance measurement is used to determine similarity, and similar training samples are stratified into the same group. Moreover, a clinical label is not required to train an unsupervised model that can be applied in more situations. In contrast, supervised learning is used when the endpoints of the treatments such as tumor control or toxicity grades are known, which requires a large amount of training samples to avoid overfitting. Unsupervised methods, such as clustering methods or the use of principal component analysis, provide means to reduce the learning problem curse of dimensionality through feature extraction, and to aid in the visualization of multivariable data and the selection of the optimal learning method parameters for supervised learning methods (48). Each method has its own merits and pitfalls (49). Deep learning is the preferred method when a large amount of data are included in the cohort. Creating a highly complex deep learning model that provides performances similar to simpler statistical tests or machine learning algorithm is redundant (50).

As mentioned earlier, a radiomics model could be validated repeatedly to confirm its potential value for clinical application. Generally, external validation is considered to be a stronger test for a model than an internally validated prediction model because it produces more credible and robust results (51). Many methods have been used successfully to evaluate the performance of radiomics models; the receiver-operating characteristic (ROC) curve is the method most commonly utilized for discrimination analysis and the concordance index is usually used for validation of survival analysis (52).

### Radiogenomics Approach

A radiogenomics study may be exploratory or hypothesis-driven. In exploratory studies, a common approach is multiple hypotheses testing, whereby the features extracted are tested against a mass of genomic variables. Accurate conclusions can be reached from exploratory analyses but statistical correction to the significance level is required. The false discovery rate is the optimal metric for controlling the expected proportion of “discovery” that is false when conducting multiple comparisons. Furthermore, hierarchical cluster analysis has proved to be a useful tool for exploratory analysis of gene expression data, which is an algorithm that groups similar objects into clusters that are distinct from each other. Moreover, a type of tree diagram, known as a dendrogram, is usually used to show hierarchical relationships between different clusters. In contrast, when using the hypothesis-driven approach, researchers collect a sufficient number of imaging phenotypes and then investigate them with a specific hypothesis in mind. For example, Konstantinidis et al. used this method in a prospective clinical trial and confirmed a previous hypothesis that MRI can act as an imaging biomarker for prediction of the response to chemotherapy in patients with unresectable intrahepatic cholangiocarcinoma (ICC) (53).

### Current Application of Radiogenomics in Oncology

Radiogenomics takes advantage of big data analysis approaches that explore meaningful information for decision-making in the diagnosis and treatment of cancer (54). Furthermore, radiogenomics provides an in-depth understanding of tumor biology and captures imaging biomarkers with relevant implications. These approaches have been validated in a variety of tumors (55). Here we summarize the known and potential imaging features of corresponding genotypes in various types of tumors and their value and feasibility in clinical practice.

### Glioblastoma

Glioblastoma multiforme (GBM) is considered to be the most common life-threatening brain cancer, accounting for 45% of primary central nervous system tumors with an average overall survival of only 15 months (56, 57). This dismal prognosis is mainly due to the invasiveness of the tumor, which responds variably to treatment, and the infiltrative ability of tumor cells that cannot be detected with the current imaging technologies. Heterogeneity exists not only at the patient level but also at the level of a single tumor, indicating that GBM includes a wide range of genetic abnormalities and regional transformations in response to microenvironmental cues (58). In general, the most reliable diagnostic imaging method is MRI because of its excellent soft tissue contrast (59). With progress in the genetic understanding of GBM, multiple strategies are being developed to associate the radiological features of GBM with genomic phenotypes, for prediction of the therapeutic response and clinical prognosis.

GBM also shows biological heterogeneity and includes proneural, neural, classical, and mesenchymal subtypes (60). Studies have demonstrated that imaging-based biomarkers not only allow prognostic stratification of individual patients but also have an important role in disease diagnosis (61–63). For example, Zinn et al. (64) identified a causal link between radiomic texture features and periostin expression levels, an important gene involved in GBM invasion and recurrence (65). The results provide evidence for the potential use of non-invasive interventions as predictors of disease prognosis in future clinical trials (66).

#### IDH Mutation

One of the best known molecular biomarkers in GBM development is the mutation status of isocitrate dehydrogenase (IDH) 1/2 (67). This enzyme is found to regulate the citric acid cycle (68) and increase angiogenesis (69). A retrospective study of 176 patients with GBM conducted in Korea (70) revealed a significant association between the MRI features and corresponding genomic profiles, demonstrating that these imaging characteristics can be used to predict *IDH* mutation status. Specifically, this study found that a higher proportion of insular involvement, larger tumor volumes, a higher volume ratio on T2-weighted and contrast-enhanced T1-weighted images (solid enhancing portion on the contrast-enhanced T1-weighted volume), and a higher apparent diffusion coefficient (ADC) were more prevalent in patients with *IDH* mutation. Similarly, Mazurowski et al. (63) analyzed the imaging data of 110 patients with lower-grade gliomas from The Cancer Genome Atlas (TCGA). They found a strong association between a quantitative feature, angular standard deviation (ASD), which measures irregularity of the tumor boundary, and the IDH-1p/19q subtype (p < 0.0017). Higher ASD is generally considered a predictor of poorer outcomes.

#### ATRX Loss

The alpha thalassemia/mental retardation X-linked gene (*ATRX*) is involved in chromatin remodeling and maintenance of telomeres. *ATRX* mutations are mainly associated with diffuse astrocytomas and gliomas with higher sensitivity to treatment. Tumors with loss of *ATRX* have been shown to a great extent to harbor a sharper hypersignal intensity area margin and a higher ADC value of the T2 hyperintense lesion compared with tumors that contain wild-type *ATRX*, which suggests a better prognosis in patients with this GBM subtype (70).

#### TP53 Mutations

*TP53* is an important gene that suppresses tumorigenesis by inducing cell cycle arrest and is frequently altered in diffuse gliomas and particularly in astrocytomas. Mutation of *p53* results in proliferation and invasion of tumor cells, which is a prognostic marker for diffuse glioma. Preoperative MRI examinations found a specific correlation of p53 with the tumor location and enhancement pattern in lower-grade glioma. Li et al. (61) indicated that Maximum_6 and Median_6 values (signals of microvessel counts on T2-weighted images) are higher in tumors with mutant than in those with wild-type p53. Furthermore, they showed that Uniformity_4, a radiological parameter indicating the consistency of the image, could predict the mutation status of *p53* (61). This observation may reflect the fact that p53 mutation increases the aggressiveness and heterogeneity of a tumor, leading to disparity of uniformity.

#### O6-Methylguanine-DNA-Methyltransferase Methylation

The association between epigenomic clusters and MRI traits was also uncovered by research that created predictive machine learning-based classification models. The status of DNA methylation using O6-methylguanine-DNA-methyltransferase (MGMT) promoter methylation status and the tumor’s copy number variation profile can be used to classify glioblastoma in various subgroups (71). Due to the function of MGMT in promoting DNA repair and reducing the efficacy of alkylating events, epigenetic silencing of the MGMT DNA repair gene through promoter methylation leads to irreparable DNA damage and cell death and increased sensitivity to alkylating chemotherapy.

In a study, MGMT methylation was mainly observed in tumors with a higher percentage of contrast-enhancing tumor volume to complete tumor volume, higher Gaussian-normalized relative cerebral blood volume (nrCBV) and nrCBV in the contrast-enhanced and total tumor volumes (72). The indicator relative cerebral blood volume (rCBV) is widely utilized and can reflect tumor hypoxia and angiogenesis, which can be evaluated more precisely by imaging of vessel size. The methylated MGMT promoter is also related to the presence of pseudoprogression. Therefore, increases in enhancement within 3 months after completion of radiotherapy in patients with MGMT methylation are regarded as treatment-related effects (pseudoprogression) rather than progressive disease. Tixier et al. (73) investigated the combination of the MGMT status with radiomics and found that a feature named edge descriptor was significantly correlated with MGMT methylation and predicted better survival of GBM patients.

#### Phosphatidylinositol 3-Kinase -Akt-Mammalian Target of Rapamycin Pathway

Identification of a marked correlation between expression of the mammalian target of rapamycin (mTOR) and the maximum rCBV in the enhanced GBM (62) has paved the way for prediction of mTOR status from images. Given that mTOR inhibitors can improve the response of GBM to temozolomide, this prediction model may facilitate identification of a suitable patient population.

Furthermore, Cui el at has shown that the high-risk volume (HRV) was higher in GBMs with mutations in either Nuclear Factor I (*NF1)* or *PIK3CA* than in those that were wild type (72). These two genes play a critical role in the progression of GBM. It has been shown that mutations of *NF1*, a tumor suppressor gene, are quite common in the mesenchymal molecular subtype, which has a very poor prognosis due to aggressive biological behavior (60, 74). Patients with GBM who have an activated phosphatidylinositol 3-kinase (PI3K) signaling pathway also have poorer outcomes than those who do not (75). Inhibitors targeting the PI3K pathway are under active development and offer hope for patients with GBM.

#### Epidermal Growth Factor Receptor Amplification

Another study (76) identified compelling imaging connections for six oncogenes or tumor suppressor genes (*EGFR*, *PDGFRA*, *PTEN*, *CDKN2A*, *RB1*, and *TP53*) in 48 biopsies collected from 13 tumors. By establishing multivariate predictive models for each gene, the investigators found a significant association between amplification of *EGFR* and local binary patterns texture on rCBV maps.

Apart from a single gene mutation, advanced high-throughput measurement of, for example, a change in mRNA expression and DNA copy number variation could also enable identification of correlations between individual genes or loci and particular imaging features. Jamshidi et al. (77) created a multilevel radiogenomics association map to highlight genes that showed concordant mRNA expression and gene dose changes and their links with MRI features. That study identified 34 gene loci, including *LTBP1*, *RUNX3*, and *KLK3*, as biomarkers of GBM.

### Breast Cancer

Breast cancer is the most common malignancy in women and is regarded as a heterogeneous and complex disease. Breast cancer can be classified into luminal A, luminal B, human epidermal growth factor receptor 2 (HER2), and basal molecular subtypes (78). Specific molecular subtypes are shown to have different patterns of initial disease presentation, different relapse-free survival rates, and distinct variations in response to treatment. Conventional imaging techniques, including mammography, ultrasound, and MRI, are used to detect malignant lesions and monitor disease progression.

#### Gene Expression

Women with *BRCA1/2* gene mutation are considered as being at a higher risk of developing breast and/or ovarian cancer (79). Li et al. (80) found that computerized mammographic assessment of breast density and parenchymal patterns (phenotypes of coarseness and contrast) from radiographic texture analysis could together be used to distinguish *BRCA1/2* gene-mutation carriers from low-risk women.

#### Molecular Subclassification

Several studies have attempted to delineate the correlation between findings on breast MRI and molecular subtype. For example, Grimm et al. (81) identified two dynamic imaging features that were independent predictors of the luminal A and luminal B subtypes: 1) the ratio of enhancement of the tumor to that of the fibroglandular tissue at two time points; 2) the sequence number at which peak enhancement occurs. Meanwhile, Blaschke et al. (82) found that HER2-positive cancers showed more rapid early uptake of contrast compared to other subtypes, and Mazurowski et al. (83) demonstrated that the imaging features of luminal B had a higher tumor enhancement ratio. Moreover, Zhu et al. (84) developed three deep learning models to distinguish between breast cancer subtypes by analyzing dynamic contrast-enhanced MRI scans. However, they found that the best area under the curve of the models was only 0.65, indicating that deep learning can help the research of radiogenomic correlations, but is still limited.

In another study, the MRI phenotype with a heterogeneous enhancement pattern was proven to be significantly associated with immune-related genes characterizing the interferon-rich subtype, most of which is part of triple-negative breast cancer (85). In 10 patients with breast cancer, radiogenomics analysis showed that 12 dynamic contrast-enhanced MRI-specific traits were significantly associated with high expression of immune-related genes, including *STAT1*, *CXCL9*, and *IFIT1* (85).

#### Signaling Pathways

The tumor necrosis factor-alpha (TNF-α)/NF-kappaB/Snail pathway is one of the critical molecular pathways in breast cancer and is involved in many activities related to the tumor cell, including epithelial-mesenchymal transition (86), proliferation (87), angiogenesis (88, 89), invasion, and metastasis (90). Wu et al. analyzed 10 quantitative imaging characteristics related to enhancement patterns in the tumor-adjacent region and found an association between the TNF signaling pathway and parenchymal imaging features in breast cancer, which are of prognostic value (91).

Janus kinases (JAK) belong to the family of non-receptor tyrosine kinases and centrally involved in activation of signal transducer and activator of transcription proteins (STAT) proteins in breast cancer (92). The JAK/STAT pathway is a rapid cytoplasmic to nuclear signaling pathway and leads to the activation of genes through a process called transcription (93). Disrupted JAK-STAT signaling could induce carcinogenesis. Yeh et al. (94) intended to perform quantitative radiomic analysis on 47 invasive breast cancers and obtained gene expression data on corresponding fresh frozen tissue samples. Gene set enrichment analysis was used to identify significant associations between the 186 gene pathways and the 38 image-based features. As a result, they found that tumors with higher expression of JAK/STAT and VEGF pathways appeared to have positive correlation with contrast, difference variance, and entropy, and negative correlation with homogeneity and image linearity.

#### RNA Sequencing

The Oncotype Dx Recurrence Score (ODxRS), which incorporates the mRNA expression of 21 genes, is used clinically to predict the prognosis of early-stage invasive breast cancer (95). Sutton et al. (96) aimed to illustrate the relationship between ODxRS and morphological and texture-based image features extracted from MRI imaging of 95 breast cancer patients. Two MRI-derived image features, kurtosis and histologic nuclear grade, were found to be significantly correlated with the ODxRS.

Long noncoding RNAs, defined as the noncoding transcript, are a crucial group of regulatory RNAs that have been implicated in the development of numerous types of solid tumors. The emergence of next-generation sequencing technologies has provided a good opportunity for increasingly rapid development of RNA sequencing, shedding light on novel and undiscovered transcriptional and epigenetic regulators in breast cancer. Yamamoto et al. (97) performed a radiogenomics analysis to determine the association between the enhancing rim fraction and the expression of 14,880 long noncoding RNAs. Interestingly, the enhancing rim fraction score was found to be correlated with a known predictor of tumor metastasis, HOTAIR (homeobox transcript antisense intergenic RNA). These findings prompted the development of radiogenomics in breast cancer, which has the potential to become an alternative to genetic testing.

### Renal Cell Carcinoma

Renal cell carcinoma (RCC) constitutes 2%–3% of all cancers in adults worldwide and has an increasing incidence (98). Clear cell RCC (ccRCC) is the most common subtype and accounts for 70%–80% of all RCCs, followed by papillary RCC and chromophobe RCC (99). Percutaneous biopsy is widely used for preoperative diagnosis of RCC; however, its use is controversial because of potential complications and sampling errors. Recently, radiomics techniques that focus on changes at the molecular level have become an effective way of screening quantitative features for accurate diagnosis of these tumors and prognostic assessment.

#### Von Hippel–Lindau Mutation

Previous studies have shown that loss or mutation of Von Hippel-Lindau tumor suppressor (*VHL)* is a critical driver of ccRCC and is believed to occur at an early stage in renal cancer (100). According to our current understanding of the tumorigenesis of ccRCC, alteration of *VHL* promotes the expression of hypoxia-inducible factors, which are believed to be the central event in upregulation of angiogenesis-related factors. There is mounting evidence of radiogenomic associations between subtype-discriminative CT features and *VHL* mutation status, possibly arising from a previous finding of significant associations between ccRCC with *VHL* mutation and clear tumor margins, nodular enhancement, and an intratumoral vasculature on enhanced CT images (101). Li et al. (102) developed a radiomics model with all eight minimum redundancy maximum relevance features from CT images of 170 RCC patients and found it to be significantly associated with *VHL* mutation.

#### Polybromo 1 Mutation

The second most frequent mutation in ccRCC is in Polybromo 1 (*PBRM1*), which, like *VHL* mutation, becomes mutated in the early stage of tumorigenesis. A meta-analysis that included 2942 patients reported that a lower PBRM1 expression level is correlated with a dismal prognosis, advanced clinical stage, and a higher Fuhrman nuclear grade in ccRCC as well as responsiveness to immune checkpoint inhibitors (103). Kocak et al. found that high-dimensional quantitative CT texture analysis was potentially able to identify ccRCC with and without *PBRM1* mutations using the artificial neural network (ANN) and random forest (RF) algorithms as machine learning classifiers; RF outperformed ANN in that study (95.0% vs 88.2%) (104).

#### Runt-Related Transcription Factor-3 Methylation

The runt-related transcription factor-3 (*RUNX3*) gene, a noted tumor suppressor gene, regulates gene expression in some dominant developmental pathways and has antitumor activity in various types of tumors (105). *RUNX3* is reportedly inactivated in numerous types of tumors and is involved in various biological tumor processes, including epithelial-mesenchymal transition, adhesion, migration, and invasion. Therefore, the methylation level of *RUNX3* can also influence tumor cell phenotype. For example, Cen et al. (105) reported a significant relationship between a high level of *RUNX3* methylation and shorter survival. Meanwhile, high intratumoral vascularity, unclear margins, and a left-sided tumor can be used to predict high *RUNX3* methylation level (106).

#### BRCA1-Associated Protein 1 Mutation

The *BAP1* (BRCA1-associated protein 1) gene mutation, which is present in 15% of ccRCCs, has been associated with Fuhrman grade 3 or 4 tumors and poor survival, as well as greater sensitivity to radiotherapy and mTOR blockade (107). Shinagare et al. (108) identified ill-defined margins and the presence of calcification to be critical predictors of *BAP1* mutation in patients with ccRCC.

### Liver Cancer

One of the most aggressive malignancies is primary liver cancer, the most common types of which are hepatocellular carcinoma (HCC) and ICC (109). HCC is the most clinically prevalent subtype and is characterized by high morbidity and mortality rates worldwide (110). Over the past several decades, there is strong evidence of a link between HCC and chronic hepatitis B virus (HBV) infection (111). At present, several imaging modalities for HCC screening/surveillance and diagnosis are endorsed by the international guidelines, including ultrasonography, CT, and MRI, which can provide essential information about tumor staging and are used to assess the treatment response. So far, there have been few radiogenomics studies in HCC.

Early in 2007, Kuo et al. (112) performed a radiogenomics analysis to mine the relationship between imaging features in HCC and expression of 313 liver-specific genes. *CYP27A1* and *CYP4V2*, which belong to the cytochrome p450 superfamily, were found to be responsible for drug metabolism and detoxification and to be significantly associated with the tumor margin score. In a series of studies, Banerjee et al. identified a CT biomarker called radiogenomic venous invasion, which was found to be a strong predictor of microvascular invasion in HCC (113). Moreover, the presence of radiogenomic venous invasion has been associated with tumor recurrence and a shorter survival time (113). Xia et al. (114) reported several methodological benefits of the association between CT imaging features and gene expression profiles when deciphering non-invasive surrogate biomarkers for HCC. They constructed different gene modules according to their prognostic significance and identified enrichment of the MEred gene module in the biological functions and pathways involved in virus-related RNA transcription that were significantly associated with the determined prognostic geometry features. For example, hepatitis B can greatly increase the risk of liver cirrhosis and HCC. Furthermore, functional enrichment of the MEyellow gene module promotes lipid metabolism and complement activation. Interestingly, an earlier study demonstrated alterations in fatty metabolism in HCC that could promote dedifferentiation of tumor cells. Miura et al. (115) retrospectively performed clinicopathological and global gene expression analyses and found that *SLCO1B3* was upregulated in HCC cases with a higher intensity lesion in the hepatobiliary phase on gadolinium-ethoxybenzyl-diethylenetriamine pentaacetic acid-enhanced MRI.

Taouli et al. (116) analyzed dozens of qualitative and quantitative imaging traits seen in preoperative CT or MRI data and found some to be correlated with aggressive genomic signatures of HCC. For example, the “infiltrative pattern” showed the strongest associations with gene signatures representing enhanced cellular proliferation and vascular invasion while “presence of macrovascular invasion” was also an important imaging feature that showed a significant correlation with the molecular signatures of vascular invasion, distant metastases, and TNM staging in HCC.

ICC is a relatively rare but lethal primary liver cancer originating from the intrahepatic bile duct epithelium. ICC has high expression levels of EGFR and vascular endothelial growth factor gene (VEGF) as well of pro-angiogenic and hypoxia markers. Sadot et al. (117) investigated the relationship between imaging phenotypes and molecular profiling of ICC by visually analyzing imaging features and performing texture analysis with immunohistochemical assessment of molecular markers in 25 patients with ICC. Linear regression analysis showed that the correlation texture feature was significantly associated with expression of VEGF, whereas correlation and entropy texture features were significantly related to expression of EGFR.

### Colorectal Cancer

Colorectal cancer (CRC) is the third most common cancer worldwide and is characterized by substantial spatial phenotypic and genotypic variations (118). The development of colon cancer involves multiple steps with a continuous cumulative effect of genetic mutation in tumor suppressors and oncogenes. CT and MRI, as well as ^18^F-FDG-PET imaging, are widely used for the diagnosis, monitoring of therapeutic response, and prognosis of CRC (119). Recently, there has been an increasing number of investigations on whether or not conventional imaging techniques can predict critical gene mutations in CRC without the need for an invasive procedure.

#### KRAS Mutation

Mutation of the *KRAS* gene is found in nearly two fifths of CRCs and is regarded as an independent prognostic factor for survival and a downstream marker of tumor resistance to anti-EGFR-targeted therapy. Lubner et al. (120) found that skewness, a texture parameter that can measure asymmetry of the pixel histogram on CT, showed a negative correlation with *KRAS* mutation status. Furthermore, Shin et al. (121) demonstrated that a higher prevalence of *KRAS* mutations was significantly associated with a more advanced nodal stage and the presence of polypoid tumors. Rectal cancers with *KRAS* mutations have a higher axial tumor length and a larger ratio of axial to longitudinal tumor dimensions on rectal MRI. Miles et al. (122) analyzed multiparametric PET-CT imaging phenotypes using a recursive decision-tree to integrate measurements of ^18^F-FDG uptake, CT texture, and perfusion. This methodology identified *KRAS* mutations with high accuracy and a low false-positive rate. However, Chen et al. (123) found that an increased accumulation of FDG measured using a 40% threshold level for maximal uptake of CT-based tumor width was an independent predictor of *KRAS* mutations.

#### Other Gene Mutations

A preliminary study (124) that sought to identify other frequent gene mutations in CRC found a significant correlation of tumor location with *APC* and *RASA1* mutation, a significant association of absence of lymph node metastasis with *BRCA2* mutation, and a correlation of tumor size with *FLT4* mutation, as well as a higher frequency of *ATM* mutation in patients with a positive circumferential resection margin. However, the results of multiple comparisons were not significant. Therefore, large-scale studies are needed for additional evaluation and to validate these early observations.

### Gastric Cancer

Gastric cancer is one of the most common and aggressive solid tumors worldwide and has its highest incidence and mortality rate in Eastern Asia (125). Approximately 20%–40% of patients who receive standard treatment develop recurrent disease (126). Based on the gene expression profile of gastric cancer, there are four molecular subtypes: Epstein-Barr virus-positive, microsatellite unstable, chromosomal instability (CIN), and genomically stable (127). Previous studies have shown that the CIN subtype of gastric cancer has a distinct prognosis. For example, Sohn et al. found that patients with the CIN subtype obtained the greatest benefit from adjuvant chemotherapy (128). CT is regarded as the routine preoperative evaluation modality. Furthermore, Lai et al. (129) investigated the relationship between CT imaging features and CIN status and found that an acute tumor transition angle was the most accurate imaging feature of the CIN subtype of gastric cancer, which provides additional prognosis-related information.

### Lung Cancer

Lung cancer is another common cancer with a high mortality rate and accounts for 13% of all newly diagnosed cancers (98). Histologically, lung cancer can be divided into non-small cell lung cancer (NSCLC) and small cell lung cancer (SCLC). Nearly 85% of patients with lung cancer have NSCLC (130).

NSCLC is a group of distinct diseases with genetic and cellular heterogeneity due to different mutations in oncogenic signaling pathways. Conventional imaging methods include radiography and CT, which can provide valuable information for diagnosis, clinical staging, and treatment decisions. Invasive biopsy plays a central role in the pathological diagnosis; however, only a small portion of tissue is generally obtained and cannot completely reflect the properties of the whole tumor. Therefore, radiogenomics mapping is being increasingly used to solve the growing demand for prognostic image-based biomarkers.

There is an urgent need to identify high-risk patients who are more likely to relapse and require more extensive follow-up or aggressive treatment. Invasion of lung cancer into the visceral pleura is a frequent pathological phenomenon (131). Tumors with visceral invasion are classified as T2a and have a bleak prognosis. Lee et al. (132) defined a quantitative pleural contact index, which is the ratio of the tumor-pleura contact length to the maximum tumor length on CT images, and investigated its prognostic value as well as the molecular background of pleural invasion. They found that the pleural contact index was associated with remodeling of the extracellular matrix and that related genes also acted as independent predictors of overall survival in patients with NSCLC. Fave et al. (133) calculated the radiomic features from images of 107 patients with stage III NSCLC and found that texture-strength measured at the end of treatment significantly predicted the risk of local recurrence.

Zhou et al. (134) established a radiogenomics map that integrated CT imaging features and gene expression profiles in patients with NSCLC. By summarizing gene functional enrichment and CT characteristics, they identified a cluster of co-expressed genes involved in the epidermal growth factor pathway that had a significant relationship with the degree of ground-glass opacity and irregular nodules or nodules with poorly defined margins.

#### BRAF Mutation

BRAF is a serine/threonine-protein kinase that belongs to the Ras/mitogen-activated signaling pathway family. *BRAF* mutations occur in 2%–5% of NSCLCs; they are responsible for phosphorylation of MEK and ultimately promote cell proliferation and survival (135). Halpenny et al. compared the CT features of *BRAF*-mutated lung carcinomas with those of lung carcinomas with wild-type *BRAF* and found no significant difference between BRAF lesions and non-BRAF lesions (136).

#### Nuclear Factor Kappa-Light-Chain-Enhancer of Activated B-CellActivation

Nuclear factor kappa-light-chain-enhancer of activated B-cells (NF-ĸB) is an important transcription factor that regulates multiple signaling cascades and is a positive mediator of cell growth and proliferation (137). Therefore, NF-ĸB and its target genes are involved in the process of tumorigenesis. Nair et al. (138) found a relationship between NF-ĸB expression and tumor glucose metabolism on FDG-PET that could be a potential prognostic biomarker.

#### Epidermal Growth Factor Receptor Mutation

EGFR belongs to a family of receptor tyrosine kinases and is expressed in more than 60% of NSCLCs (139). Most EGFR mutations involving exons 18, 19, and 21 are considered to predict a favorable response to treatment with an EGFR tyrosine kinase inhibitor (140). Gevaert et al. (141) confirmed that ground-glass opacities and nodule margins are indicative of the presence of EGFR mutations. Aerts et al. (142) collected high-resolution CT imaging of 47 patients with early-stage NSCLC before and after gefitinib therapy to investigate if radiomics can identify a gefitinib response-phenotype. Changes in features between two scans, delta volume and delta maximum diameter, were strongly predictive of EGFR mutation status and of the associated gefitinib response.

#### Anaplastic Lymphoma Kinase Rearrangement

The *ALK* gene encodes a tyrosine kinase transmembrane protein, a member of the superfamily of insulin receptors, which are responsible for 3%–7% of NSCLCs and often undergo fusion with echinoderm microtubule-associated protein-like 4 (143). Crizotinib and two other ATP-competitive ALK inhibitors, ceritinib and alectinib, have achieved improved outcomes in this subset of patients. A meta-analysis (144) that summarized the imaging features from 12 studies that included 2,210 patients with NSCLC found that the presence of ALK rearrangement in a primary tumor showed distinct imaging characteristics, including a likelihood of being solid and being less likely to show cavitation and air bronchograms.

### Ovarian Cancer

Ovarian cancer is the deadliest malignancy of the female genital tract and has five major histopathological subtypes. Ninety percent of ovarian cancers are high-grade serous ovarian cancer (HGSOC), which has the least favorable prognosis (145). Previous studies have demonstrated the genomic complexity and heterogeneity of ovarian cancer (146). Genetic heterogeneity, including copy number variant, transcriptome analysis, and methylation array, has been discovered in HGSOC, which may explain its drug resistance and open up new avenues for targeted molecular-based treatment. CT is an indispensable imaging examination for patients with HGSOC and can allow staging evaluation for preoperative planning and determination of surgical resectability. Several studies have shown that the CT features can predict critical molecular alteration events in HGSOC, which may have substantial prognostic and therapeutic implications at the time of diagnosis (147).

#### BRCA Mutation

Approximately 15%–20% of HGSOC cases are inherited. *BRCA1* and *BRCA2* mutations are the most commonly identified germline mutations, whereas 6% of these tumors harbor somatic *BRCA* mutations (148). Previous cohort studies have determined that *BRCA1/2* mutations are associated with improved long-term survival in patients with ovarian cancer (149, 150). A retrospective study assessed the preoperative CT scans of 108 patients with HGSOC and found qualitative CT features that could distinguish the *BRCA* mutation status of HGSOC. Multiple regression analysis showed that the pattern of peritoneal disease, presence of peritoneal disease in the gastrohepatic ligament, and supradiaphragmatic lymphadenopathy were associated with HGSOC harboring *BRCA* mutation, whereas the presence of peritoneal disease in the lesser sac and left upper quadrant, mesenteric involvement, and lymphadenopathy in the supradiaphragmatic and suprarenal para-aortic regions were correlated with wild-type *BRCA* (147).

#### Inter-Site Heterogeneity

Inter-site heterogeneity describes a phenomenon whereby tumor cells from different metastatic sites in the same patients can show distinct morphological and phenotypic characteristics. This phenomenon is found in ovarian cancer and is informative for the prognosis and treatment decisions. Vargas et al. (151) provided valuable data indicating that CT texture-based measures can be utilized to evaluate spatial heterogeneity across multiple metastatic lesions and to predict clinical outcomes in patients with HGSOC. In particular, patients with amplification of the cyclin E1 gene exhibited more inter-site texture heterogeneity on CT imaging.

#### BRAF Mutation

Recent studies have demonstrated mutations of *BRAF* or *KRAS* in approximately 60% of serous borderline tumors and low-grade serous carcinomas (152). The presence of *BRAF* mutation in a serous borderline tumor is a favorable prognostic factor and may inhibit progression to low-grade serous cancer (153). A retrospective study by Nougaret et al. showed that the presence of bilateral ovarian masses, peritoneal lesions, and higher solid tumor volumes was significantly associated with wild-type *BRAF* (154).

### Prostate Cancer

Prostate cancer is the most prevalent malignancy in men in the United States. An epidemiological investigation in 2015 showed that 1.6 million men were diagnosed with prostate cancer and that there had been a 66% increase in its incidence over the previous decade (155).

Currently, the National Comprehensive Cancer Network risk stratification system, which is mainly based on pathological grading from a biopsy sample, prostate serum antigen levels, and T staging, is widely used (156). Even though its prognostic precision has been reproduced in various settings, numerous studies have shown that the impact of adverse pathology is unavoidably underestimated in about 38%–46% of patients (157), partly because of the spatial heterogeneity in tumor growth patterns. Imaging examination can overcome the sampling bias resulting from prostate biopsy; therefore, the properties of the entire tumor can be assessed using a non-invasive platform. Multiparametric MRI is the most accurate imaging modality for detection and localization of prostate cancer lesions and provides both functional tissue information and anatomical information (158). Stoyanova et al. (159) first identified a significant association between quantitative multiparametric MRI features and gene expression in multiparametric MRI-guided biopsy samples. The identified gene clusters related to radiomic features were used for gene ontology analysis and were correlated with distinct biological processes, including immune response, metabolism, cell, and biological adhesion.

#### PTEN Deletion

The *PTEN* gene encodes for the phosphatase and tensin homolog and is a tumor suppressor gene on chromosome 10 in region 10q23 that is mutated or deleted throughout the human cancer spectrum (160). Deletion of *PTEN* has been confirmed to be an important event in prostate carcinogenesis due to activation of the PI3K/Akt signaling pathway. Furthermore, loss of *PTEN* has been shown to confer a seven-fold increased mortality risk from prostate cancer (161). McCann et al. (162) analyzed the preoperative multiparametric MRI scans of 45 peripheral zone cancer foci and found weak correlations of the reverse reflux rate constant between the extracellular space and the plasma and of the Gleason score with *PTEN* expression in prostate cancer. However, further investigation and validation of this finding is needed.

### Retinoblastoma

Retinoblastoma originates from immature retinal cells. It is the most prevalent intraocular malignancy in children, with 95% of cases diagnosed by the age of 5 years. Most bilateral tumors are caused by germline mutations in the *RB1* gene whereas the majority of unilateral retinoblastomas are associated with the presence of somatic *RB1* mutations (163). Furthermore, amplification of *MYCN* was identified in wild-type RB1 retinoblastomas, suggesting that amplification of this gene can trigger tumorigenesis in the background of a functional retinoblastoma protein. Jansen et al. (164) assessed the association between imaging features and the genome-wide mRNA expression profiles of 60 patients with retinoblastoma and found a correlation between a lower photoreceptor gene signature and advanced-stage imaging features, including multiple lesions and a large eye size. Moreover, expression of *MYCN* was associated with subretinal seeding, while differential expression of *SERTAD3* was significantly associated with diffuse growth, a plaque-shape, and multifocality.

### Head and Neck Squamous Cell Cancer

Head and neck squamous cell carcinoma (HNSCC) is the sixth most common cancer worldwide (165). The Cancer Genome Atlas (TCGA) has revealed that human papillomavirus-associated tumors are accompanied by *PIK3CA* mutations, loss of *TRAF3*, and amplification of *E2F1*, whereas smoking-related HNSCCs exhibit a higher frequency of *TP53* mutations and *CDKN2A* copy number alterations. Furthermore, mutations of the chromatin modifier *NSD1* and the Wnt pathway genes *AJUBA* and *FAT1* were also detected in a subgroup of HNSCCs (166). Zwirner et al. (167) followed a hypothesis-driven approach for finding associations between radiomic heterogeneity and genetic aberrations and found that *FAT1* somatic mutations were associated with reduced radiomic measures of tumor heterogeneity, possibly clarifying the reason for the previously described better prognosis of patients with human papillomavirus-negative, *FAT1*-mutated HNSCC.

### Unresolved Issues/Limitations

Convincing evidence has emerged showing that there is a moderate association between imaging characteristics and genomic or related characteristics of various kinds of cancers (**Table 1**). However, adoption of this work into common clinical practice needs to overcome significant challenges.

**Table 1:** Specifications of radiomic studies in different cancers.

Studies				Study type	No. of specimen	Inclusion criteria	No. and type of Radiomic features	Image Modality	Clinical Characteristics	Statistical analysis
**Brain cancer**										
Li et al. (61)			Retrospective study		Validation: 84 from TCGA 272(training:validation= 182:92)	Grade II or III glioma	431 (intensity, shape, texture, and wavelet)	T2-weighted MRI	p53 status	Gene ontology (GO) analysis, LASSO Cox regression, SVM classifier, ROC curve analysis
Liu et al. (62)			Retrospective study		41 patients	II or III glioma, GBM	_	MRI	ki-67, TP53 and IDH mutation, EGFR amplification, mTOR activation	hazards regression, Cox proportional hazards model
Mazurowski et al. (63)			Retrospective study		110 patients (TCGA)	Grade	5(shape)	FLAIR sequence MRI	IDH mutation, 1p/19q co-deletion	Univariate Cox proportional
Zinn et al. (64)			Retrospective study		93 patients (TCGA/TCIA/REMBR/ANDT)	GBM	310 (intensity, shape, texture, and wavelet)	MRI	Periostin expression	LASSO Cox regression
Hong et al. (70)			Retrospective study		176 patients	GBM	_	MRI	IDH 1/2 mutation, ATRX loss, MGMT promoter methylation	Univariate/multi-variate analysis, Cox regression
Kickingereder et al. (71)			Retrospective study		152 patients	GBM	31 (intensity, shape, texture, and wavelet)	MRI	Global DNA methylation subgroups, MGMT promoter methylation status, and CDKN2A loss, EGFR amplification	Univariate analysis, stochastic gradient, boosting machine, random forest, penalized logistic regression classifiers
Cui et al. (72)			Retrospective study		108 patients (TCIA)	GBM	High-risk volume (HRV)	MRI	MGMT methylation status, NF1 and PIK3CA mutation	Cox regression analysis,
Hu et al. (76)			Exploratory study		48 tissue of 13 patients	GBM	256 (240 MRI-texture features + 16 raw features [mean, SD])	MRI	Image-guided biopsy	Univariate/multi-variate analysis, decision-tree models, chi-square test
Jamshidi et al. (77)			Retrospective study		23 patients	GBM	6(contrast enhancement, necrosis, contrast-to-necrosis ratio, infiltrative versus edematous T2 abnormality, mass effect, subventricular zone involvement)	MRI	messenger RNA expression, DNA copy number variation (CNV)	global gene set enrichment approach, gene set enrichment analysis, Pearson correlation algorithm
**Breast cancer**										
Li et al. (80)	Retrospective study				453	breast cancer	Coarseness, contrast, percent density, radiographic texture analysis	full-field digital mammograms	BRCA1/2 mutation	Pearson correlation algorithm, ROC analysis
Grimm et al. (81)	Retrospective study				275 patients	breast cancer	56 (size and shape, gradient, texture, dynamic)	DCE MRI	ER, PR, HER2 status	binary multivariate, logistic regression model
Mazurowski et al. (83)	Retrospective study				48 patients	breast cancer	23 (morphologic, textural, dynamic)	MRI	ER, PR, HER2 status	logistic regression, likelihood ratio tests
Zhu et al. (84)	Exploratory study				270 patients	breast cancer	45-56	DCE MRI	ER, PR, HER2 status	off-the-shelf deep features approach, three neural network structures
Yamamoto et al. (97)	Retrospective study				70 patients	breast cancer	47 (geometric, statistical, spatiotemporal)	DCE MRI	ER, PR, p53, HER2 status, lncRNA transcripts	Cox regression analysis, log-rank Mantel-Cox test
**Renal cell carcinoma**										
Karlo et al. (101)	Retrospective study				233 patients	Clear cell RCC	8 quantitative features	CT	VHL, PBRM1, SETD2, KDM5C, or BAP1 genes	Fleiss k, Fisher exact test, t test
Li et al. (102)	Retrospective study				255 patients	Clear cell RCC	156	CT	VHL mutations	random forest based wrapper algorithm(Boruta),Wilcoxon rank-sum test
Kocak et al. (104)	Retrospective study				45 patients (TCGA)	Clear cell RCC	828 (first-order, texture, and wavelet)	CT	PBRM1 mutation	artificial neural network (ANN) algorithm, random forest
Cen et al. (106)	Retrospective study				106 patients	Clear cell RCC	9 (tumor architecture margin, intratumoral calcifications, collecting system invasion, necrosis, renal vein invasion, enhancement, gross evidence of intratumoral vascularity, long diameter	CT	RUNX3 methylation level	chi-square test, univariate/multivariate logistic regression analysis
Shinagare et al. (108)	Retrospective study				103 patients	ccRCC	size, margin, composition, necrosis, growth pattern, and calcification	CT	mutational status (VHL, BAP1, PBRM1, SETD2, KDM5C, and MUC4)	Pearson’s v2 test, the Mann-Whitney U test
**Liver cancer**										
Kuo et al. (112)	Retrospective study				30	HCC	6 (Internal arteries, texture heterogeneity, Wash-in_max, Washout_maxi, Necrosis, Tumor margin score)	CT	cDNA microarray analysis (8,364 genes)	Hierarchical clustering, false discovery rates analysis
Xia et al. (114)	Retrospective study				371 patients	HCC	37(intensity, geometry, and texture)	CECT	RNA Seq	Cox’s proportional hazard model, Spearman rank correlations.
Miura et al. (115)	Retrospective study				77 patients	HCC	4	EOB-MRI	RNA Seq	Mann-Whitney U test,
Taouli et al. (116)	Retrospective study				39 patients	HCC	11+4 (size, enhancement ratios, wash-out ratio, tumor-to-liver contrast ratios)	CECT	13 HCC gene signatures	logistic regression analysis
Sadot et al. (117).	Retrospective study				25 patients	ICC	13 (first-order, texture)	CT	hypoxia markers (EGFR, VEGF, CD24, P53, MDM2, MRP-1, HIF-1α, CA-IX, and GLUT1)	univariate analysis test, multiple linear regressions
**Colorectal cancer**										
Vlachavas et al. (119)	Retrospective study				30 patients	CRC	kinetic parameters k1, k2, k3 and k4 and influx	18F-FDG PET	RNA-seq samples	Spearman correlation analysis, LASSO Cox regression
Lubner et al. (120)	Retrospective study				77 patients	hepatic metastatic CRC	6	CECT	tumor grade, baseline serum CEA, KRAS mutation	Cox proportional hazards models
Shin et al. (121)	Retrospective study				275 patients	Rectal cancer	22 (intensity, geometry, and texture)	MRI	KRAS mutation	chisquared test, Wilcoxon rank-sum test, Fisher’s exact test
Miles et al. (122)	Retrospective study				33 patients	CRC	3 (SUVmax, MPP, blood flow)	18F-FDG PET	HIF-1α score, KRAS mutation, MCM-2	recursive decision-tree, Monte Carlo analysis
Chen et al. (123)	Retrospective study				103 patients	CRC	7	18F-FDG PET	TP53, KRAS, APC, BRAF, and PIK3CA alteration	Mann-Whitney U test, logistic regression analysis
Horvat et al. (124)	Retrospective study				65 patients	RC	34 (intensity, textures)	MRI	APC, TP53, KRAS,CRM, ATM,BRCA2	Fisher’s Exact test, the K-means algorithm, univariate analysis
**Lung cancer**										
Lee et al. (132)		Retrospective study			117+88 patients	Stage I NSCLC	6	CT	gene expression microarray data	Spearman correlation coefficient, Cox proportional hazard regression model
Zhou et al. (134)		Retrospective study			113 patients	NSCLC	35 (shape, margin, texture, tumor environment, and overall lung characteristics)	CT	RNA sequencing	the Spearman correlation metric, Pearson correlation coefficients, univariate Cox proportional hazards regression
Halpenny et al. (136)		Retrospective study			188 patients	NSCLC	15	CT	BRAF mutation	Cochran Mantel-Haenszel test, logistic regression
Nair et al. (138)		Retrospective study			355 patients	NSCLC	_	18F-FDG PET	NF-ĸB mutation	Student’s t-test or Wilcoxon test, Cox proportional hazards (CPH) analysis
Gevaert et al. (141)		Retrospective study			186 patients	NSCLC	85	CT	EGFR and KRAS mutations	decision tree modeling, univariate analysis
**Ovarian cancer**										
Nougaret et al. (147)	Retrospective study				108 patients	HGSOC	16 qualitative features	CT	BRCA mutation	logistic regression, Cox proportional hazards regression
Vargas et al. (151)	Retrospective study				38 patients	HGSOC	12 inter-site tumor heterogeneity texture metrics	CT	cyclin E1 gene (CCNE1)	LASSO regression
Nougaret et al. (154)	Retrospective study				59 patients	SBT LGSC	3	CT	KRAS and BRAF hot-spot mutations	univariate/multivariate logistic regression analysis
**Prostate cancer**										
Stoyanova et al. (159)	Retrospective study				37 patients	grade 2 prostate tumor	49 features	MRI	3 prognostic signatures (Polaris Cell Cycle Progression, Decipher, Genomic Prostate Score)	Pearson correlation distances, two-way hierarchical clustering, GO analysis
McCann et al. (162)	Retrospective study				45 foci of 30 patients	peripheral zone prostate cancer	3 groups: DWI-based, T2-weighted, and DCE-MRI-based image features.	MRI	PTEN expression	Spearman rank correlation coefficient
**Other tumors**										
Lai et al. (129)	Retrospective study				40+18 (testing+validation)	Gastric cancer	14 qualitative and 2 quantitative imaging traits	CT	Chromosomal instability status	the chi-square or Fisher’s exact test,Mann-Whitney U test, logistic regression
Jansen et al. (164)	Retrospective study				60 patients	retinoblastoma	25 imaging features	MRI	PAX2, MYCN mutation	generalized linear modeling
Zwirner et al. 167	Retrospective study				20 patients	HNSCC	3	CT	TP53, FAT1 and KMT2D	Mann-Whitney U test, robust linear regression

IDH, isocitrate dehydrogenase; LASSO, least absolute shrinkage and selection operator; SVM, support vector machine; FLAIR, ﬂuid-attenuated inversion recovery; EGFR, epidermal growth factor receptor; mTOR, mammalian target of rapamycin; DCE-MRI, dynamic contrast material enhanced MRI; RCC,clear cell renal cell carcinoma; HCC, hepatocellular carcinoma; ICC, intrahepatic cholangiocarcinoma; CRC, colorectal cancer; MPP, mean value of the tumor pixels with positive values; SAM, Significance Analysis of Microarrays; NSCLC, nonsmall cell lung cancer; LGSCs, low-grade serous carcinomas; SBTs, serous borderline tumors; HGSOC, high-grade serous ovarian cancer; HNSCC, head and neck squamous cell carcinoma.

The foremost limitation of current radiogenomics models is their repeatability and reproducibility (168). Researchers should not overlook the variability arising from use of different equipment and different software or that arising at different clinics. These problems lead to results that are difficult to reproduce, which has largely impeded the progress of radiogenomics models. Therefore, implementation of standard practice guidelines is a critical step to ensure the accuracy and reliability of analytic results in radiogenomics studies (169).

First, differences in acquisition radiomics parameters and variations in contrast enhancement protocols due to different machines and patient conditions are major issues during image acquisition and reconstruction. Therefore, establishment of standardized protocols for each modality is an essential step to avoid this situation. Second, reproducibility and reliability are crucial in the tumor segmentation process. The variability among different readers in delineation of ROIs depends on the methods of segmentation used. It has been shown that semi-automatic delineation not only has machine-like precision, but also can be manually corrected. With regard to feature extraction, a wide range of voxel intensities and image noise needs to be filtered to preserve the desirable signal and reduce unwanted noise; variation in discretization methods also leads to different results. A suitable solution would be to adopt absolute discretization with fixed bin sizes, which have better repeatability and stability (170). Finally, feature nomenclature, algorithms, methodology, and software have also varied between the different studies, which can jeopardize the accuracy and performance of the models (52). Therefore, the lack of conformity between the above aspects must be elucidated and unified to eliminate differences as much as possible in the process of feature extraction.

On the other hand, there are still some shortcomings in the design and construction of radiogenomics studies. Firstly, most studies are retrospective with small sample sizes and a lack of prospective validation cohorts. The main restriction for deep learning radiogenomics is the limited size of the available datasets. The insufficiency of the required volume of data can lead to inadequate stratification (171–173) among training, validation and testing datasets, compromising the model adaptation, optimization, and evaluation process, respectively. In addition, quantitative descriptors with interpretability are also important in clinical practice. Therefore, interpretable models combined with open-access, curated and high-quality public benchmark databases with complete genomic and imaging data across disease types are urgently needed. Only in this way can we perform better investigations to address tumor heterogeneity. All these deficiencies are prone to produce statistical issues related to overfitted data and multiple testing.

Another drawback is the lack of multicenter studies, which raises doubts that the findings to date would be reproducible by difference in readers, imaging equipment, and radiologists in different fields. Due to the technological imperfections, there is a significant mismatch between the perceived capabilities and the actual capabilities of artificial intelligence in current studies.

### Future Direction

To eliminate the inconsistency and uncertainty of different studies, the most critical matter is to formulate a standardized workflow and internationally agreed methods to guide the implementation of efficient studies. Several consortia have been developed worldwide for this purpose. One is the Image Biomarker Standardization Initiative (IBSI) (35), which was established to reach consensus and provide a standard for calculation of the frequently used radiomics features and for the image processing needed before extraction of the radiomics features. It shall also provide guidelines for summarizing comprehensive information on radiomics experiments (174). The TRIPOD (Transparent Reporting of a multivariable prediction model for Individual Prognosis Or Diagnosis) statement is a guideline for reporting studies with development or validation of a multivariable prediction model (175). Generally, the standardization of radiomics methods is a prerequisite for subsequent clinical translation, and the workflow and benchmarked values defined by the noted consortia, represent advancement to calibrate future investigations of radiogenomics.

Some additional details on the quality of radiomics studies are being developed, notably for standardization of radiogenomics protocols and quality assurance. High-quality research is key to progress in this field. Researchers should insist on following the “FAIR (findability, accessibility, interoperability, and reusability) guiding principles” by ensuring that all research objectives are traceable, accessible, interoperable, and recyclable, thus enabling independent validation and quality assurance (176). In the near future, international cooperative efforts will be required to confirm the added value of promising quantitative models compared to existing methods (177). Concerted efforts are required to provide a thorough understanding of the relationship between dataset sizes, possible confounders, and the performance of outcome prediction. Consequently, large-scale multicenter prospective studies are needed to generate machine learning-based models.

Fusing imaging modalities (24) with no *a priori* knowledge or evidence about their optimal combination for the targeted therapy can lead to unnecessary, redundant analysis with a negative effect on the final decision. Thus, clinicians should provide insight and participate in cooperation with the data science engineers regarding specific lesion attributes concerning the followed diagnosis protocols. Other types of clinical information, including laboratory exam results, anthropometricorphic (height and weight), demographic (age and sex) and supplementary imaging modalities can introduce diversity and complementarity toward achieving better problem formulation, improved predictive power, and a robust decision support process (178).

Moreover, future advances in imaging technology, post-processing techniques, and computer-aided diagnostic technology, including sophisticated functional imaging techniques such as ^23^Na-MR imaging, chemical exchange saturation transfer (CEST), blood oxygen level-dependent (BOLD) MRI, and hybrid PET-MRI, may reinforce the role of radiogenomics in tumor classification and treatment. Development of a pool of labeled metabolites has triggered further insight into cellular activity and provides a potential tool for identification of correlations between imaging features and tumor genotype.

## Conclusion

In conclusion, radiogenomics is an inevitable outcome following the trend of precision medicine nowadays. With three main advantages, including lower cost than conventional genome sequencing, availability of whole tumor information as opposed to a limited biopsy specimen, and increased spatial resolution, a comprehensive radiomics-based approach may reflect the spatial variation and heterogeneity of voxel intensities within a tumor and generate predictive and prognostic information (179). Applying a mass of automatic extraction of characterized data algorithms and combining them with clinical information into open databases, radiogenomics has emerged as a bridge between the phenotype and genotype of tumors (180).

It is expected that the role of radiogenomics will extend to every aspect of cancer management, from calibrating detection and diagnosis to predicting the therapeutic response, to risk surveillance. Furthermore, input of imaging data directly into the discovery engine rather than using radiomics feature sets previously developed or recognized and constructing a customized sequence through deep learning architecture has encouraged further exploratory radiogenomics studies (14).

In the future, it is expected that the data acquired from imaging examinations will be transformed into quantitative data and interfaced with existing databases to offer diagnostic and prognostic evidence for supporting clinical decision-making.

## Authors’ Contributions

LS and HR contributed to the writing of the paper. XY, JL, and ZC provided essential assistance and designed the constructure of this review. YC, HZ, and PS supervised the manuscript and revised the paper. The authors read and approved the final manuscript. All authors contributed to the article and approved the submitted version.

## Conflict of Interest

The authors declare that the research was conducted in the absence of any commercial or financial relationships that could be construed as a potential conflict of interest.

## Abbreviations

ADC, apparent diffusion coefficient; ALK, anaplastic lymphoma kinase; ATRX, alpha thalassemia/mental retardation X-linked gene; BAP1, BRCA1-associated protein 1; BOLD-MRI, blood oxygen level-dependent MRI; CBV, cerebral blood volume; ccRCC, clear cell renal cell carcinoma; CEST, chemical exchange saturation transfer; CIN, chromosomal instability; CNNs, convolutional neural networks; CRC, colorectal cancer; EGFR, epidermal growth factor receptor; EML4, echinoderm microtubule-associated protein-like 4; FDG, fluorodeoxyglucose; GBM, glioblastoma multiforme; GCGMM, GrowCut with cancer-specific multiparametric Gaussian Mixture Model; HBV, hepatitis B virus; HCC, hepatocellular carcinoma; HGSOC, high-grade serous ovarian cancer; HNSCCs, head and neck squamous cell cancers; HOTAIR, homeobox transcript antisense intergenic RNA; HRV, high-risk volume; IBSI, Image Biomarker Standardization Initiative; ICC, intrahepatic cholangiocarcinoma; IDH, isocitrate dehydrogenase; KRAS, Kristen rat sarcoma viral oncogene; MGMT, O6-methylguanine-DNA-methyltransferase; MRI, magnetic resonance imaging; NF-ĸB, nuclear factor kappa-light-chain-enhancer of activated B-cells; NSCLC, non-small cell lung cancer; PET-CT, positron emission tomography-computed tomography; RCC, renal cell carcinoma; ROC, receiver-operating characteristic; ROI, region of interest; RUNX3, runt-related transcription factor-3 gene; SCLC, small cell lung cancer; SPECT, single-photon emission-computed tomography; TNF-α, tumor necrosis factor-alpha; TRIPOD, Transparent Reporting of a multivariable prediction model for Individual; Prognosis Or Diagnosis.
